# Triptolide Modulates TREM-1 Signal Pathway to Inhibit the Inflammatory Response in Rheumatoid Arthritis

**DOI:** 10.3390/ijms17040498

**Published:** 2016-04-02

**Authors:** Danping Fan, Xiaojuan He, Yanqin Bian, Qingqing Guo, Kang Zheng, Yukun Zhao, Cheng Lu, Baoqin Liu, Xuegong Xu, Ge Zhang, Aiping Lu

**Affiliations:** 1 Institute of Basic Research in Clinical Medicine, China Academy of Chinese Medical Sciences, Beijing 100700, China; zgzyfdp@126.com (D.F.); hxj19@126.com (X.H.); gqq5432@163.com (Q.G.); zhengkang13@126.com (K.Z.); zhyk_87@163.com (Y.Z.); lv_cheng0816@163.com (C.L.); 2 Institute for Advancing Translational Medicine in Bone & Joint Diseases, Hong Kong Baptist University, Hong Kong, China; 3 E-Institute of Chinese Traditional Internal Medicine, Shanghai University of Traditional Chinese Medicine, Shanghai 201203, China; xiaobian504@126.com; 4 School of Life Science and Engineering, Southwest Jiaotong University, Chengdu 610031, China; 5 Zhengzhou Hospital of Traditional Chinese Medicine, Zhengzhou 450002, China; liubqmm@163.com

**Keywords:** Triptolide, rheumatoid arthritis, TREM-1 signal pathway, bioinformatics analysis

## Abstract

Triptolide (TP), an active component isolated from *Tripterygiumwilfordii Hook F*, has therapeutic potential against rheumatoid arthritis (RA). However, the underlying molecular mechanism has not been fully elucidated. The aim of this study is to investigate the mechanisms of TP acting on RA by combining bioinformatics analysis with experiment validation. The human protein targets of TP and the human genes of RA were found in the PubChem database and NCBI, respectively. These two dataset were then imported into Ingenuity Pathway Analysis (IPA) software online, and then the molecular network of TP on RA could be set up and analyzed. After that, both *in vitro* and *in vivo* experiments were done to further verify the prediction. The results indicated that the main canonical signal pathways of TP protein targets networks were mainly centered on cytokine and cellular immune signaling, and triggering receptors expressed on myeloid cells (TREM)-1 signaling was searched to be the top one shared signaling pathway and involved in the cytokine and cellular immune signaling. Further *in vitro* experiments indicated that TP not only remarkably lowered the levels of TREM-1 and DNAX-associated protein (DAP)12, but also significantly suppressed the activation of janus activating kinase (JAK)2 and signal transducers and activators of transcription (STAT)3. The expression of tumor necrosis factor (TNF)-α, interleukin (IL)-1β and IL-6 in lipopolysaccharides (LPS)-stimulated U937 cells also decreased after treatment with TP. Furthermore, TREM-1 knockdown was able to interfere with the inhibition effects of TP on these cytokines production. *In vivo* experiments showed that TP not only significantly inhibited the TREM-1 mRNA and DAP12 mRNA expression, and activation of JAK2 and STAT3 in ankle of rats with collagen-induced arthritis (CIA), but also remarkably decreased production of TNF-α, IL-1β and IL-6 in serum and joint. These findings demonstrated that TP could modulate the TREM1 signal pathway to inhibit the inflammatory response in RA.

## 1. Introduction

Rheumatoid arthritis (RA) is a complicated disease caused by various factors, such as environmental and genetic factors [1], and is characterized by an overactive immune system and synovial inflammation in multiple joints which will ultimately lead to irreversible joint destruction and severe disability in the absence of adequate treatment [2]. Proinflammatory cytokines and chemokines produced by synoviocytes and infiltrating immune cells are implicated in the RA disease pathogenesis [3]. In addition, many cytokines and chemokines are also found in RA patients’ synovial fluid, such as interleukin (IL)-1α, IL-3, IL-2, IL-4, IL-6, IL-8, IL-17, interferon (IFN) γ, tumor necrosis factor (TNF) β, transforming growth factor (TGF) β, granulocyte-macrophage colony-stimulating factor (GM-CSF) and macrophage inflammatory protein-3α (MIP-3α) (CCL20) [3,4]. These cytokines and chemokines are able to activate nuclear factor-κB (NF-κB), boost cyclo-oxygenase-2 (COX-2) production , induce nitric oxide synthase (iNOS), synthesis of prostaglandin E2 (PGE2) and nitric oxide (NO), which result in the inflammation in synovial accompanied by arthrosis, swelling, hyperplasia, angiogenesis, bone destruction, and finally arthritic decay [5,6,7]. Meanwhile, massive generation and integration of genomic, proteomic, signaling, and metabolomic data suggest that RA genes vary in its expression patterns [8]. These evidences implicate that RA is even more complex than initially predicted.

Currently, there are three types of therapeutic agents for RA in clinical practice: nonsteroidal anti-inflammatory drugs (NSAIDs); disease modifying antirheumatic drugs (DMARDs); steroid and biological response modifiers, and they have effects of relieving RA severity, slowing the progression of RA, and preventing the subsequent damage of joint [9]. However, the application of these therapies in clinical has been astricted as the results of their side reactions with a high frequency and high cost of treatment. Triptolide (TP), isolated from the root of *Tripterygiumwilfordii Hook F* (TW, a traditional Chinese medicine), is a diterpenetriepoxide biologically active natural product. Previous studies have showed that TP had multiple biological functions including immunosuppression, anti-inflammation and anti-cancer [10,11], and had been proved to be effective in the treatment of some inflammatory and autoimmune diseases, especially RA [12]. However, the underlying molecular mechanism of TP on RA has still not been fully elucidated.

Bioinformatics analysis technology, which can integrate multiple data and help us to better understand biological relevant processes in organic life, has become an essential way to life sciences studies. Moreover, bioinformatics analysis method can help us to search for some information about related genes, proteins, and drugs, and construct the interactional experimental system model, and then, enable the discovery and exploration of molecular interaction networks visualization [13]. Therefore, the purpose of this research is to explore the mechanism of TP on RA in global through combining drug-target prediction, network analysis and experimental validation.

## 2. Results and Discussion

### 2.1. Results

#### 2.1.1. Triggering Receptors Expressed on Myeloid Cells (TREM)-1 Signaling Was an Important Signaling Pathway of Triptolide (TP) on Inflammatory Response in Rheumatoid Arthritis (RA)

**Figure 1:**
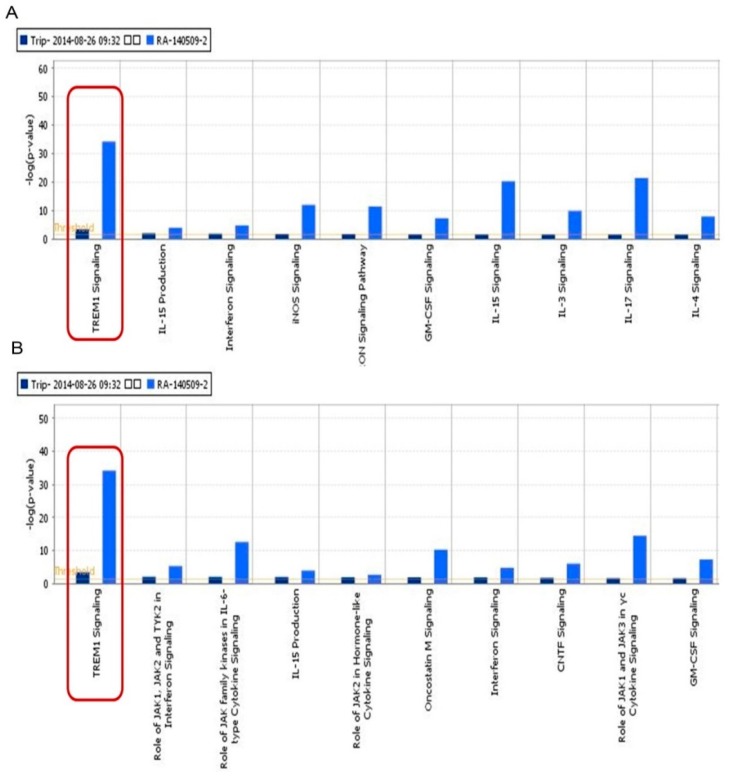
The results of bioinformatics analysis. Shared signaling pathways between gene molecular networks related with rheumatoid arthritis (RA) and protein targets molecular network of Triptolide (TP) in cytokine and cellular immune signaling performed using the Ingenuity Pathway Analysis (IPA) compare module. The signaling pathways of TP were represented as dark blue, while signaling pathways of RA were represented as light blue. (**A**) Cellular immune signaling; and (**B**) Cytokine signaling. The red boxes showed that TREM-1 signal pathway was the top one shared signaling pathway and participated in cytokine and cellular immune signaling. TREM-1: triggering receptors expressed on myeloid cells-1; IL: interleukin; iNOS: inducible nitric oxide synthase; GM-CSF: granulocyte-macrophage colony-stimulating factor; JAK: janus kinase; TYK: tyrosine kinase; CNTF: ciliary neurotrophic factor.

Eight hundred and thirty two genes related with RA were found from Gene database in NCBI. The top signaling canonical pathways related with RA genes were shown in Figure S1A. The top fifteen signaling pathways were focused on cellular immune response, cytokine signaling, humoral immune response and intercellular and second messenger signaling. Then, eight human target proteins of TP were found from PubChem database. The details are shown in Table S1. After that, the molecular networks of TP targets proteins were obtained and shown in Figure S1B, which included TREM-1 signaling, NF-κB signaling, IL-6 signaling, phosphatidylinositol 3-kinase (PI3K)/protein kinase B (AKT) signaling, IL-17 signaling, *et al.* Based on the classification of signaling pathways in Ingenuity Pathway Analysis (IPA), we found most signaling related with TP targets were focused on cytokine and cellular immune. In canonical pathway module of IPA, three hundred and forty six and three hundred and ninety four signaling pathways of TP and RA were obtained, respectively. In addition, 214 signaling pathways were shared by TP and RA. We listed the top ten shared signaling pathways of TP and RA related to cell immune response and cytokine signaling. Further comparative analysis showed that TREM-1 signal pathway was measured to be the top one shared signaling pathway and participated in cytokine and cellular immune signaling (Figure 1A,B).

#### 2.1.2. TP Inhibited the Activation of TREM-1 Signal Pathway and Production of Inflammatory Cytokines in Lipopolysaccharides (LPS)-Induced U937 Cells

**Figure 2:**
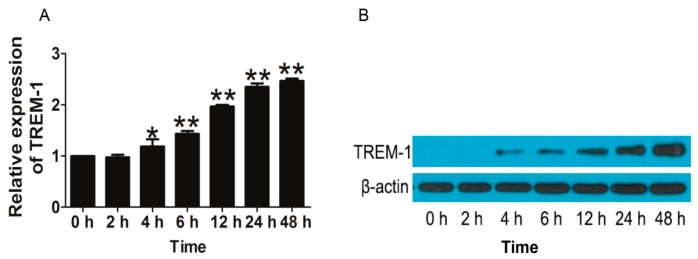
TREM-1 mRNA expression in U937 cells after lipopolysaccharides (LPS) stimulation. U937 cells were treated with 100 ng/mL of LPS for 0, 2, 4, 6, 12, 24 and 48 h, respectively. TREM-1 mRNA (**A**) and protein (**B**) levels were measured by RT-PCR and Western blots, respectively. All results are presented as mean ± standard deviation (SD) of three independent experiments, each performed in triplicate. * *p* < 0.05, ** *p* < 0.01 *versus* 0 h.

To verify the effects of TREM-1 on inflammatory reaction, U937 cells were stimulated with lipopolysaccharides (LPS) and the mRNA and protein level of TREM-1 were detected in different time points. The results of real-time PCR showed that TREM-1 mRNA level was gradually increased in a time-dependent manner (Figure 2A). Western blot results showed that TREM-1 protein expression increased from 4 h after LPS stimulation (Figure 2B). Then, to avoid any cytotoxic effects of TP, the cytotoxicity of TP was investigated. No significant cytotoxicity was observed when the cells were exposed to up to 12.5 nM TP for 24 h (Figure S2). Therefore, the concentration of TP at 12.5 nM was used for the following study.

**Figure 3:**
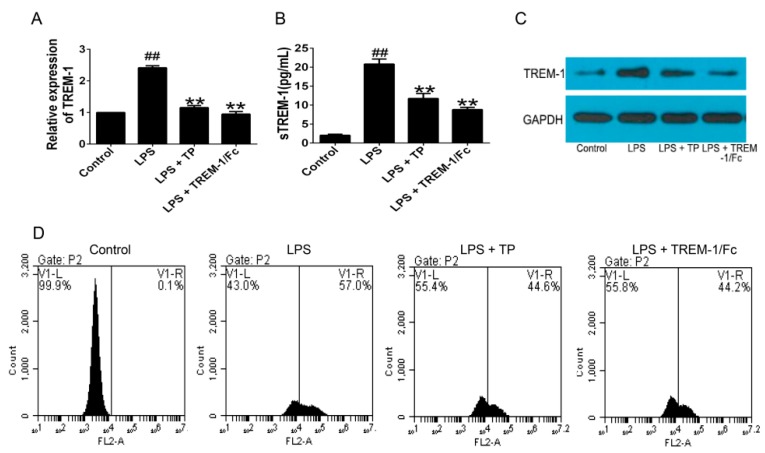

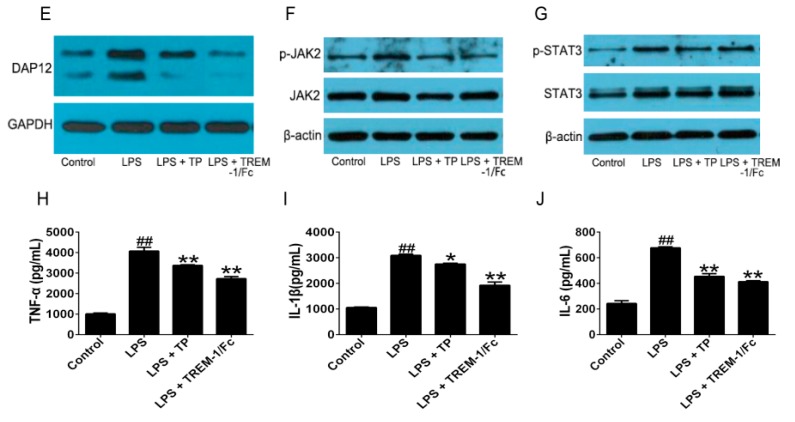
Effects of TP on TREM-1 signal pathway and inflammatory cytokines expression in LPS-induced U937 cells. (**A**) Effect of TP on TREM-1 mRNA levels in U937 cells, as measured with quantitative RT-PCR; (**B**) Effect of TP on the secretion of TREM-1, as measured with enzyme-linked immunosorbent assay (ELISA); (**C**) Effect of TP on TREM-1 protein levels in U937 cells, as measured with western blot; (**D**) Effect of TP on the expression of TREM-1 on U937 cells surface, as measured with flow cytometry; (**E**–**G**) Effect of TP on DAP12, p-JAK2, p-STAT3 protein levels in U937 cells, as measured with western blot; and (**H**–**J**) Effect of TP on the secretion of TNF-α, IL-1β, IL-6, as measured with ELISA. Data are expressed as mean ± SD from three independent experiments. ^##^ *p <* 0.01, compared to the control group. ** *p* < 0.01, * *p* < 0.05, compared to LPS group. FL2-A means the fluorescence peak area in flow cytometric analysis.

In order to observe whether TP can influence the expression of TREM-1, TREM-1 mRNA and protein levels were detected in the TP-treated U937 cells. TREM-1 expression on U937 cells surface was also detected. The results showed that TREM-1 mRNA and protein levels were significantly decreased in TP-treated U937 cells compared with the LPS model group (Figure 3A–C). Consistent with these results, TREM-1 expression on U937 cells surface was also significantly inhibited by TP (Figure 3D). Furthermore, expression of DAP12 and phosphorylation of janus activating kinase (JAK) and signal transducers and activators of transcription (STAT)3 were suppressed by TP (Figure 3E–G). Finally, to detect the effect of TP on proinflammatory cytokines, we measured the TNF-α, IL-1β and IL-6 levels in supernatant of TP-treated U937 cells. As shown in Figure 3H–J, LPS potently induced TNF-α, IL-1β and IL-6 production in U937 cells, and TP could remarkably inhibit the production of these cytokines.

#### 2.1.3. TREM-1 Signal Pathway Might Participate in the Regulatory Effect of TP on LPS-Induced Inflammatory Cytokines Production

**Figure 4:**
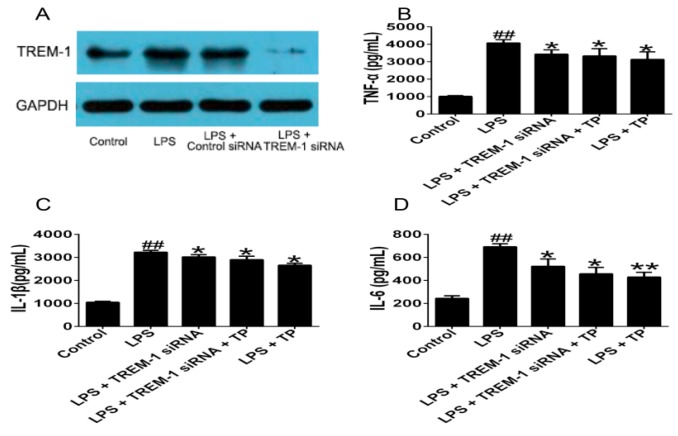
Effects of TP on inflammatory cytokines expression after TREM-1 knockdown. PMA-induced U937 cells were treated with or without 1 μg/mL LPS, and then transfected with TREM-1 siRNA or control siRNA, respectively. After 48 h, cells were treated with or without TP for another 48 h. (**A**) TREM-1 mRNA protein levels were determined by Western blot; and (**B**–**D**) TNF-α, IL-1β and IL-6 production were determined by ELISA. ^##^ *p* < 0.01 *versus* the control group; * *p* < 0.05 and ** *p* < 0.01 *versus* the group with LPS. The data are presented as the means ± standard deviation from three independent experiments.

To determine the role of the TREM-1 signal pathway in the regulatory effect of TP on LPS-induced inflammatory cytokines production, we used TREM-1 siRNA to knockdown the expression of TREM-1 in U937 cells induced by LPS. Our results showed that the expression of TREM-1 protein was significantly decreased in phor-bol 12-myristate 13-acetate (PMA)-induced U937 cells transfected with TREM-siRNA compared with untransfected cells or cells transfected with control siRNA (Figure 4A). TREM-1 knockdown could decrease the levels of TNF-α, IL-1β and IL-6. TP treatment could still decrease TNF-α, IL-1β and IL-6 expression in LPS-induced U937 cells after TREM-1 knockdown. However, there was no significant differences between cells transfected with TREM-1 siRNA and cells transfected with TREM-1 siRNA in the presence of TP (Figure 4B–D).

#### 2.1.4. TP Alleviated the Severity of Arthritis

**Figure 5:**
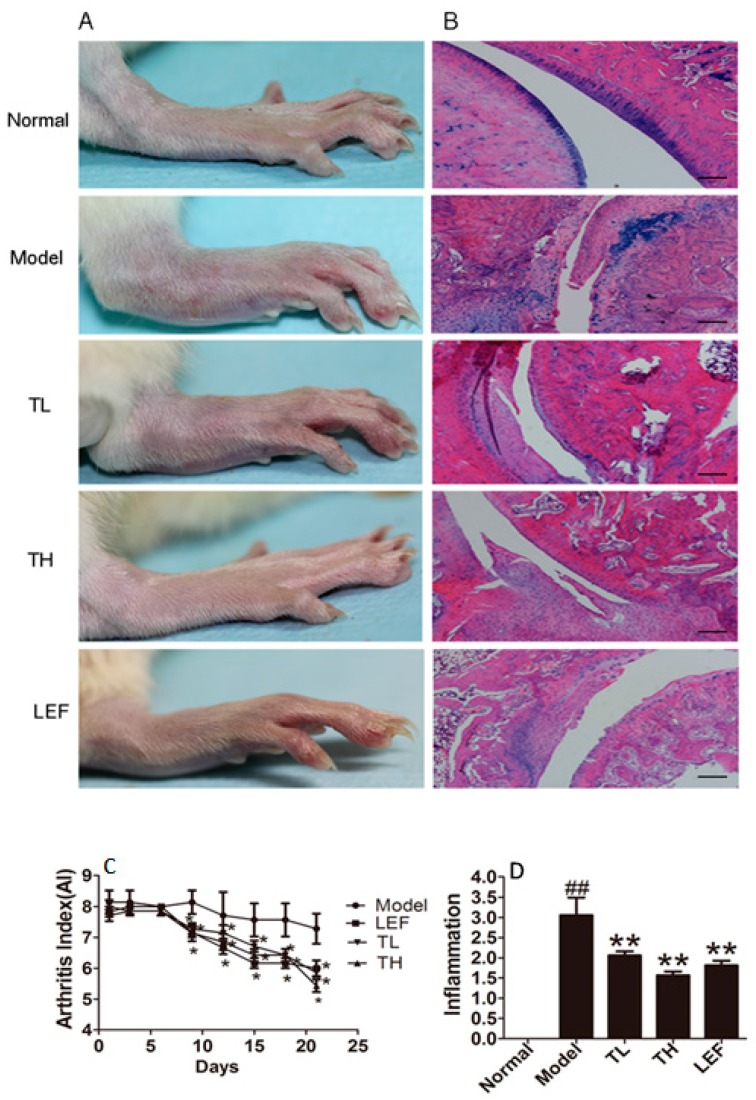
Effects of TP on collagen-induced arthritis (CIA) rats. (**A**) Morphological features of representative ankle joints; (**B**) Histopathological features of representative ankle joints haematoxylin-eosin (HE) staining, ×20, scale bars = 100 μm; (**C**) Arthritic score in different days after treatment with TP; (**D**) Inflammation scores in different groups after treatment with TP, as described in methods. TL represented for TP low dose (9.31 µg/kg) group, TH represented for TP high dose (18.62 µg/kg) group, LEF represented for Leflunomide group. Date are represented as the mean ± SD (*n* = 7), ^##^ *p* < 0.01, comparison with the normal group, * *p* < 0.05, ** *p* < 0.01, comparison with the model group.

To further research the effect of TP on the progression of arthritis, we used the collagen-induced arthritis (CIA) model in SD rats. As shown in Figure 5A,C, treatment with TP could alleviate the severity of arthritis. From day 9 after the treatment, the arthritic scores of rats treated with TP at TH (TP high dose), TL (TP low dose) and LEF (Leflunomide group) dosages were decreased. We then analyzed the histopathological changes of the ankle joints in rats from each group. As shown in Figure 5B,D, the inflammatory cells infiltration, the hyperplasia of synovial as well as cartilage and bone destruction in CIA rats were clear. Whereas, TP treatment could alleviate those histopathological changes, which showed a potential therapeutic effect of TP on CIA rats.

#### 2.1.5. TP Suppressed TREM-1 Signal Pathway in Ankle Joints of Collagen-Induced Arthritis (CIA) Rats

**Figure 6:**
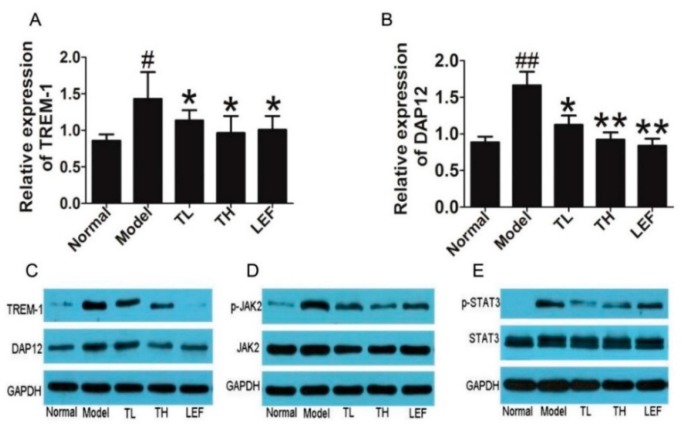
Effects of TP on the expression of TREM-1 and DAP12 in ankle joints of CIA rats. (**A**,**B**) TREM-1 and DAP12 mRNA levels in the ankle joints of CIA rats after treatment with TP; (**C**) Protein expression of TREM-1 and DAP12 in the ankle joints of CIA rats; (**D**) Expression of p-JAK2 in the ankle joints of CIA rats; and (**E**) Expression of p-STAT3 in the ankle joints of CIA rats. Date are represented as the mean ± SD (*n* = 7), ^#^ *p* < 0.05, ^##^ *p* < 0.01, in comparison with the normal group; * *p* < 0.05, ** *p* < 0.01, comparison with the model group.

Further, we investigated the TREM-1 and DAP12 levels in ankle joints of CIA rats. Our results showed that TP markedly decreased the mRNA and protein levels of TREM-1 and DAP12 (Figure 6A–C). Phosphorylation of JAK2 and STAT3 were also significantly suppressed by TP treatment (Figure 6D,E).

#### 2.1.6. TP Reduced the Expression of Proinflammatory Cytokines in Serum and Ankle Joints of CIA Rats

**Figure 7:**
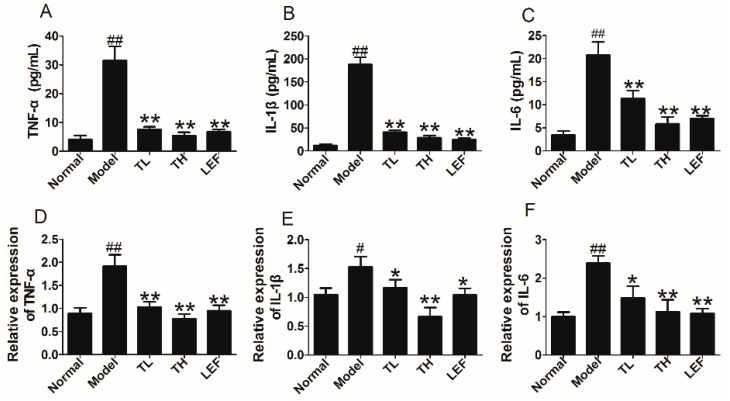
Effects of TP on proinflammatory cytokines expression in CIA rats. (**A**–**C**) TNF-α, IL-1β and IL-6 levels in serum of CIA rats after treatment with TP; and (**D**–**F**) TNF-α, IL-1β and IL-6 mRNA levels in ankle joints of CIA rats after treatment with TP. Data are represented as the mean ± SD (*n* = 7), ^#^ *p* < 0.05, ^##^ *p* < 0.01 in comparison with the normal group; * *p* < 0.05, ** *p* < 0.01, comparison with the model group.

To observe the effect of TP on production of proinflammatory cytokines, we examined the expression of IL-1β, TNF-α and IL-6 in serum of CIA rats. The data indicated that TP could decrease the levels of TNF-α, IL-1β and IL-6 in serum of CIA rats (Figure 7A–C). Furthermore, the mRNA expression of TNF-α, IL-1β and IL-6 in ankle joints of CIA rats treated with TP were also significantly decreased (Figure 7D–F).

### 2.2. Discussion

TP was reported to have the effects of immunosuppression, cartilage protection and anti-inflammation *in vivo* [14,15,16,17]. Previous studies implicated that the anti-inflammatory effects of TP were achieved through inhibiting the production of NO and the expression of iNOS by blocking NF-κB activation [18,19], inhibiting the lymphocytes proliferation [20], down-regulating the production of COX-2 and PGE2 induced by TNF-α through suppressing NF-κB and MAP kinases activation [21]. In addition, other study showed that TP have the effect of regulating inflammatory reactions mediated by Toll like receptor (TLR) and could decrease the dangerous of chronic diseases, which were correlated with the exaggerated activation of TLR [22]. However, the progress of the disease is complicated, and more mechanisms should be fully elucidated. Bioinformatics methods could provide important tools for biological relevant processes in organic life. Therefore, we hope to investigate the mechanisms of TP acting on RA by bioinformatics technology as integrating network analysis. In the present study, our bioinformatics analysis found that GM-CSF signal pathway, IL-15 signal pathway, IL-15 production, Interferon signaling were related to RA, which were consistent with previous studies [23,24,25]. Further analysis indicated that TREM-1 signal pathway was measured to be the top one shared signaling pathway and involved in the signaling pathway of cytokine and cellular immune in RA.

As a member of the Ig superfamily, TREM-1 is expressed mainly on the surface of granulocytes and a subset of monocytes and neutrophils [26,27]. Although the natural ligand of TREM-1 is unknown, activation of TREM-1 using an agonistic antibody leads to activation of a cascade of intracellular events which result in inflammatory effects, such as neutrophils degranulation, phagocytosis, and the proinflammatory chemokines production including monocyte chemoattractant protein (MCP)-1, MCP-3, IL-8, and MIP-1α [28,29,30]. Furthermore, previous study has also indicated that RA patients had higher sTREM-1 levels in plasma than healthy controls, and sTREM-1 levels in RA patients plasma were associated with the measures of disease activity, suggesting that sTREM-1 in plasma could have an important role in the inflammatory progression [31]. Here, we showed that LPS increased the mRNA and protein levels of TREM-1 and subsequently, the inflammation framework of U937 cells. Whereas, TP could remarkably inhibit the production of TREM-1 and sTREM-1 in LPS stimulated U937 cells. We also learned that the expression of TREM-1 could be activated by TLR through LPS, which made TREM-1 and TLR pathways to correlate with each other and leaded to the production of proinflammatory cytokines via NF-κB pathway [31,32]. Therefore, we assumed that one of the regulation effects of TP on TREM-1 might be through cutting off the relationship between TREM-1 and TLRs, which subsequently led to the reduction of proinflammatory cytokines. However, more detailed researches are necessary to prove this hypothesis. In addition, previous studies demonstrated that the level of TREM-1 in the synovium and sTREM-1 in synovial fluid (SF) of RA patients were increased and induced the proinflammatory cytokines expression [33,34], which demonstrated that the presence of high levels of TREM-1 and sTREM-1 in synovium and SF of RA patients might have an vital effect in the development or maintenance in RA process, or both. Our experimental validation *in vivo* indicated that TREM-1 levels in serum and ankle joints of CIA rats were increased, and TP markedly decreased the expression of TREM-1 mRNA and TREM-1 protein. In addition, both TREM-1 knockdown and TP inhibit the production of TNF-α, IL-1β and IL-6 compared with LPS group. In addition, the effects of TP on these cytokines were retained after TREM-1 knockdown, but there were no significant differences between cells transfected with TREM-1 siRNA and cells which were transfected with TREM-1 siRNA and treated with TP.

It is known to all that chronic inflammatory response has been considered as the mainly protracted course of RA disease. In our study, results from bioinformatic analysis prompted that TREM-1 signaling was involved in the inflammation regulation in TP treatment RA and JAK2/STAT3 was the down-stream molecular. Previous reports indicated that the JAK/STAT signaling activation can regulate inflammation and immune response [35]. It has been reported that STAT3 can suppress expression of proinflammatory genes and production of cytokines and chemokines in macrophages [36,37,38], which were line with results of bioinformatic analysis. Our data showed that TP markedly decreased the JAK2 and STAT3 phosphorylation and subsequently, the mRNA and protein expression of TNF-α, IL-1β and IL-6, which was also validated in our *in vivo* experiment. These evidences demonstrated that TREM-1 engagement might induce the pro-inflammatory cytokines production including IL-1β, IL-6, and TNF-α [26], indicating that TREM-1 has the effect of amplifying inflammatory responses. Therefore, our results suggested that TP might modulate TREM1-JAK2/STAT3 signal pathway to inhibit inflammatory response. Certainly, some other strategies including structure prediction will be used to further discover the action mechanism of TP on RA in our next plan.

In sum, we found a new strategy by which we can search TP’s new target and the possible mechanisms. Meanwhile, the consensus between the results analyzed by bioinformatics method and previous studies suggested bioinformatics analysis method was reliable. Our finding also suggested that bioinformatics analysis method was not only suitable for finding TP’s target and the possible mechanisms but also might be appropriate for that of other drugs.

## 3. Experimental Section

### 3.1. Analysis of Molecular Networks and Signaling Pathways of TP and RA

The human target proteins of Triptolide (CID:107985) were found in PubChem platform (http://pubchem.ncbi.hlm.nih.gov), and the word “Triptolide” was searched for in the PubChem Compound. The human target genes related with RA were found in the National Center for Biotechnology Information (NCBI) Gene database (http://www.ncbi.nlm.nih.gov/gene). “Rheumatoid arthritis” was used as a key word in Gene database searching. The obtained data were saved in an excel form for the next study.

The human target proteins and genes data acquired in the first step were imported into the IPA platform. The molecules being imputed to the IPA were termed “focus molecules”. IPA generated a set of networks base on different bio-functions. Molecules were showed as nodes, and the biological relationship between two nodes was showed as an edge (line). All edges were supported by at least one reference from a textbook, from the literature, or from canonical information stored in the IPKB. Nodes were showed with diverse shapes that represented the functional class of the gene product. The networks were sorted depending on the scores enumerated by IPA and represented the significance of the molecules for the network.

The target proteins networks of TP and RA could be established. Some major information, such as top biological pathway network information, biological functions, canonical pathways and other related bio-analytical information were included. In order to study the mechanism of TP on RA, the canonical pathways analysis in IPA was accomplished by using the compare module. In addition, IPA determined the significance of the association between the focus molecules and the canonical pathways using Fisher’s exact test.

### 3.2. Cell Culture

The human leukemic U937 cells were purchased from the American Type Culture Collection (Manassa, VA, USA). The cells were grown at 37 °C in RPMI 1640 medium (GIBCO, Gaithersburg, MD, USA) supplemented with 10% fetal bovine serum (FBS) (GIBCO, Gaithersburg, MD, USA) in a humidified atmosphere of 5% CO_2_ in air.

### 3.3. Cell Viability Assay

U937 cells were divided into 96 well plates (1.0 × 10^5^ cells/mL) and firstly incubated with phor-bol 12-myristate 13-acetate (PMA) (Sigma, St. Louis, MO, USA) for 48 h in a 5% CO_2_ incubator at 37 °C to induce macrophages. Then, the cells were washed with PBS to remove remaining PMA and non-adherent cells. Next, the cells were incubated with TP (0, 6.25, 12.5, 25, 50, 100, 200 nM) for up to 45 h at 5% CO_2_ and 37 °C. 10 μL of CCK-8 reagent (Dojindo, Tokyo, Japan) was then added into each well and the cells were incubated for another 3 h. Finally, the absorbance of each well was measured at 450 nm (test wavelength) using an ELISA reader (Bio-Tek Instruments, Winooski, VT, USA).

### 3.4. Treatment of U937 Cells with TP

U937 cells were divided into 6 well plates (1.0 × 10^6^ cells/mL) and firstly incubated with PMA for 48 h at 37 °C. After being washed for three times, the cells were pretreated with or without 100 ng/mL of Lipopolysaccharides (LPS) (Sigma, St. Louis, MO, USA) for 2 h, and then incubated with 12.5 nM of TP for another 48 h. TREM-1 Fc chimera (R&D, Minneapolis, MN, USA) was used as the positive control. The supernatant was collected for ELISA, and cells were harvested for flow cytometry and western blot analysis.

### 3.5. siRNA Transfection

The TREM-1 siRNA and control siRNA were obtained from Santa Cruz Biotechnology Inc. (Delaware, CA, USA). Macrophage-like U937 cells induced with PMA were used for siRNA transfection. In addition, a random siRNA group (negative siRNA) was used as a negative control. Transfection was performed using Lipofectamine 2000 (Invitrogen Life Technologies, Carlsbad, CA, USA) according to the manufacturer’s instructions. Brifely, after 2 h of LPS-pretreated, cells were transfected with TREM-1 siRNA or control siRNA, then treated with indicated TP for 48 h, cells and serum were harvested. TREM-1 protein expressions were detected by Western blot, and TNF-α, IL-1β and IL-6 levels were determined by ELISA kit according to the manufacturer’s protocol.

### 3.6. Animals and Experimental Protocol

#### 3.6.1. Animals

Fifty male Sprague Dawley (SpD) rats (8–10 weeks old) with a mean weight of 180–200 g were obtained from Laboratory Animal Center of the Academy of Military Medical Sciences, China. Rats were housed in an appropriate environment with an air-filtering system. The rodent license of the laboratory (NO.SYXK-2010-0032) was issued by the National Science and Technology Ministry of China. All experimental procedures were approved by the Research Ethics Committee of Institute of Basic Research in Clinical Medicine, China Academy of Chinese Medical Sciences, Beijing, China (REC2014-0917).

#### 3.6.2. Induction of CIA and Assessment of Arthritis

Bovine type II collagen and complete Freund’s adjuvant (CFA) were from Chondrex (Redmond, WA, USA). Collagen-induced arthritis (CIA) rats were immunized, as previous study described [39], by intradermal injection of 100 µL of CFA containing 100 µg of Bovine type II collagen at the base of the tail with a booster immunization as the first time on day 7. Since 7 days after the booster immunization, the rats were assessed for disease severity every two days. Arthritis severity was expressed as mean arthritic index on a 0 to 4 scale according to the following criteria [40]: 0 = no edema or swelling; 1 = slight edema and erythema limited to the foot and/or ankle; 2 = slight edema and erythema from the ankle to the tarsal bone; 3 = moderate edema and erythema from the ankle to the tarsal bone; and 4 = severe edema and erythema from the ankle to the entire leg. Each hind limb was graded, and thus the maximum possible score was 8 for each rat. A rat with a score of one or above was regarded as arthritic.

#### 3.6.3. Treatment

TP, purity > 99.0%, was from Sichuan Puerfa of China. Leflunomide (LEF) was purchased from Huitian Pharmaceutical Company (Fuzhou, China). After arthritis was successfully induced (one week after boost immunization), rats were subdivided into five groups with seven rats in each group: Normal group, Model group, TP high dose (18.62 µg/kg) group (TH), TP low dose (9.31 µg/kg) group (TL), and LEF group (10 mg/kg). The routes of drugs delivery were oral administration, and the treatment was performed once a day for 21 days. The rats in the Normal and Model groups were administered with the same volume of saline [9]. After treatment, the rats were sacrificed. The serum and the hind limbs were collected for further studies.

#### 3.6.4. Flow Cytometry

The expression of TREM-1 on U937 cells surface was detected by Flow cytometry. Each sample of 0.1 mL U937 cell suspension was stained with a solution consisting of 6 μL 0.01 M PBS and 5 µL phycoerythrin (PE)-conjugated mouse monoclonal anti-human TREM-1 antibody (Bio Legend Inc., San Diego, CA, USA) for 30 min at 4 °C in dark. After washing with PBS twice, the cells were re-suspended and fixed. A FACS Caliburflow cytometer with CELLQuest software (Becton Dickinson, San Jose, CA, USA) was used to performed flow cytometric analysis.

#### 3.6.5. ELISA Analysis

The levels of sTREM-1,TNF-α, IL-1β and IL-6 in cell culture supernatant, and the levels of TNF-α, IL-1β and IL-6 in serum of rats were determined by ELISA using commercial kits, following the manufacturer’s protocol. The human and rat TNF-α, IL-1β and IL-6 ELISA kits were obtained from eBioscience (San Diego, CA, USA) and the human sTREM-1 kit was purchased from Abcam (Cambridge, UK).

#### 3.6.6. Real-Time PCR

The RNA isolation and real-time PCR assay were conducted following the previous study protocol [41]. Briefly, ankles tissue and cells were homogenized and TRIzol reagent (Invitrogen, Carlsbad, CA, USA) was used for extracting total RNA according to the manufacturer’s protocol. First-strand complementary (cDNA) synthesis was performed using each sample containing 1µg of the total RNA with the QuantiTect Reverse Transcription Kit (QIAGEN K.K., Tokyo, Japan). The specific transcripts were optimized by quantitative real-time PCR with QuantiTect SYBR Green PCR Kit (QIAGEN K.K.) and ABI 7500 real-time PCR system (Applied Biosystems, Foster, CA, USA) were used to analyze results. Gene-specific primers used were as follows: TREM-1 (AAGGCTTGGCAGAGGCTATCA as forward and TAGGGTCATCTTTCAGGGTGTACT as reverse), DAP12 (CTGGTGCTTTCTGTTCCTTCCT as forward and ATACTTCTGGCCTCTGACCCTGA as reverse), TNF-α (GGGCAGGTCTACTTTGGAGTCATTG as forward and GGGCTCTGAGGAGTAGACGATAAAG as reverse), IL-1β (CCCAACTGGTACATCAGCACCTCTC as forward and CTATGTCCCGACCATTGCTG as reverse), IL-6 (GATTGTATGAACAGCGATGATGC as forward and AGAAACGGAACTCCAGAAGACC as reverse) and GAPDH (TGGAGTCTACTGGCGTCTT as forward and TGTCATATTTCTCGTGGTTCA as reverse). Real-time PCR performed as 40 cycles for 15 s at 94 °C, 30 s at 55 °C, and 30 s at 72 °C. The date were calculated using the ΔΔ*C*_t_ algorithm and were normalized to GAPDH expression.

#### 3.6.7. Western Blot Analysis

The U937 cells treated with TP and ankle joints of CIA rats were collected for Western blot detection. The following antibodies were used: TREM-1 antibody (rabbit polyclonal antibody, dilution 1:1000, Abcam, Cambridge, UK), DAP12 antibody (rabbit polyclonal antibody, dilution 1:500, Cell Signaling Technology Inc., Danvers, MA, USA), DAP12 antibody (rabbit monoclonal antibody, dilution 1:500, Cell Signaling Technology Inc.), JAK2 and phospho-JAK2 antibody (rabbit monoclonal antibody, dilution 1:500, Cell Signaling Technology Inc.), STAT3 and phospho-STAT3 antibody (rabbit monoclonal antibody, dilution 1:1000, Cell Signaling Technology Inc.), GAPDH antibody (rat monoclonal antibody, dilution 1:20,000, Santa Cruz Biotechnology Inc., Delaware, CA, USA) and β-actin antibody (rat monoclonal antibody, dilution 1:5000, Cell Signaling Technology Inc.). All experiments were done in triplicate.

#### 3.6.8. Histological Assay

At necropsy, the right hind limbs were harvested and dissected, fixed for at least 24 h in 4% paraformaldehyde, decalcified in 10% ethylene diamine tetraacetic acid (EDTA) for up to 2 months, then embedded in paraffin. Tissue sections (5 µm) were prepared by mounted on common slides, and then stained with hematoxylin and eosin. For the inflammation of synovial, we used a scoring system previous research described by Bendele *et al.* [42], in which magnification fields were scored for the percentage of infiltrating inflammatory cells such as mononuclear, as follows: 0 = absent; 1 = mild (1%–10%); 2 = moderate (11%–50%); and 3 = severe (51%–100%).

### 3.7. Statistic Analysis

Data were analyzed with SPSS version 13.0 software (SPSS Inc., Chicago, IL, USA). Multiple comparisons were made using ANOVA test followed by Dunnett’s test. Results were expression as the mean ± SD. *p* < 0.05 was considered as statistically significant.

## 4. Conclusions

In this study, we provided an integrative analysis by combining bioinformatics analysis with experiment validation to understand the pharmacological mechanism of TP acting on RA. The results demonstrated that TP could modulate TREM1-JAK/STAT signal pathway to inhibit inflammatory response. Our results also indicated that TREM-1 might be a potential target in therapy of RA.

## Supplementary Materials

Supplementary materials can be found at http://www.mdpi.com/1422-0067/17/4/498/s1.
