# Supplementary material for: Triptolide Modulates TREM-1 Signal Pathway to Inhibit the Inflammatory Response in Rheumatoid Arthritis
Source: Int J Mol Sci. 2016 Apr 2;17(4):498. doi: 10.3390/ijms17040498 (PMC4848954; doi:10.3390/ijms17040498)
Supplement: Supplementary file 1 [file ijms-17-00498-s001.pdf]

Supplementary Materials: Triptolide Modulates TREM-1 Signal Pathway to Inhibit the Inflammatory Response in Rheumatoid Arthritis

Danping Fan, Xiaojuan He, Yanqin Bian, Qingqing Guo, Kang Zheng, Yukun Zhao, Cheng Lu, Baoqin Liu, Xuegong Xu, Ge Zhang and Aiping Lu

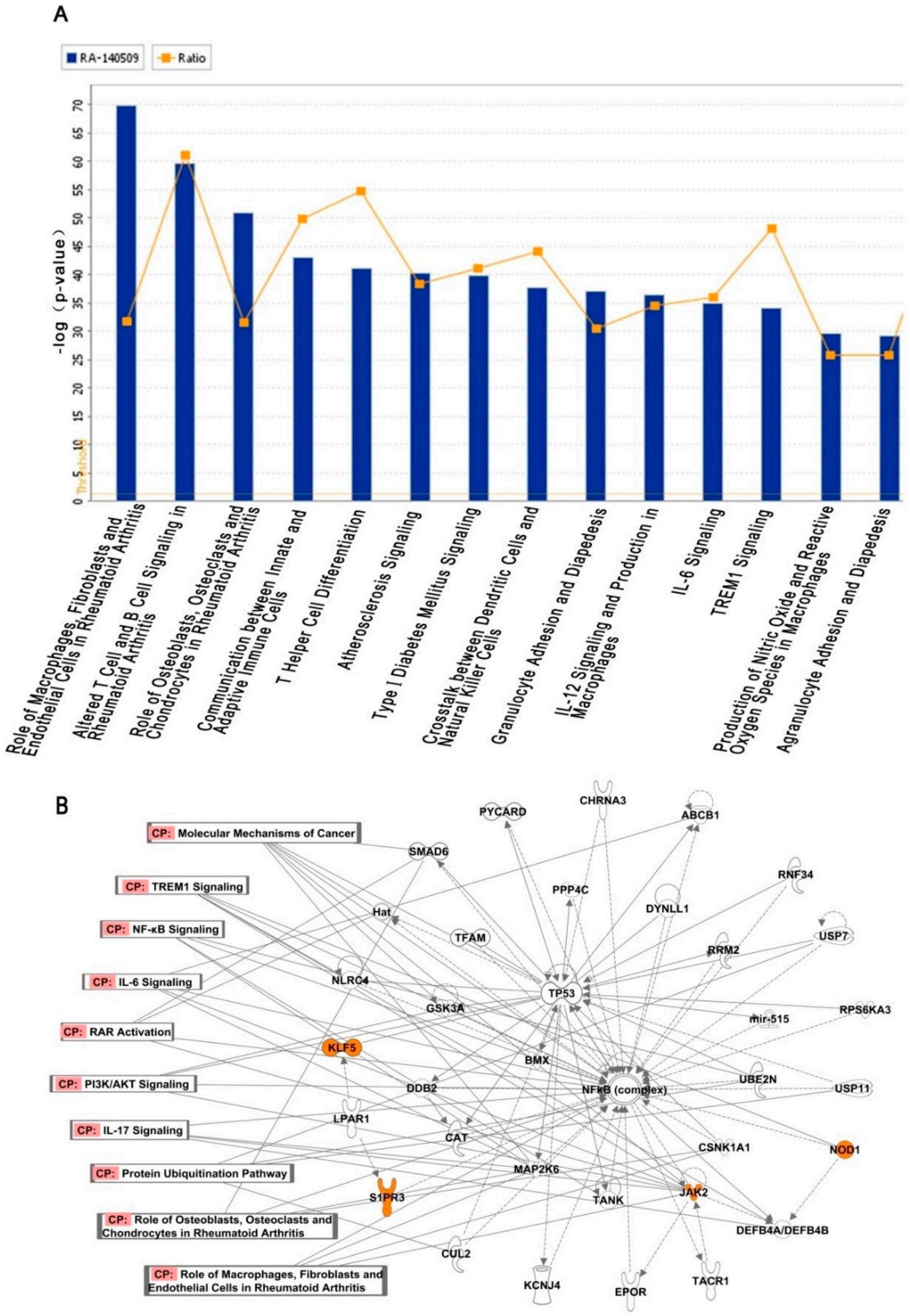

Figure S1. The results of bioinformatics analysis. (**A**) Top 15 signaling pathways over threshold related with 832 RA genes from GENE database in NCBI by Ingenuity Pathway Analysis (IPA) software; and (**B**) The molecular networks of Triptolide (TP) target proteins, Orange nodes represented the protein targets of TP.

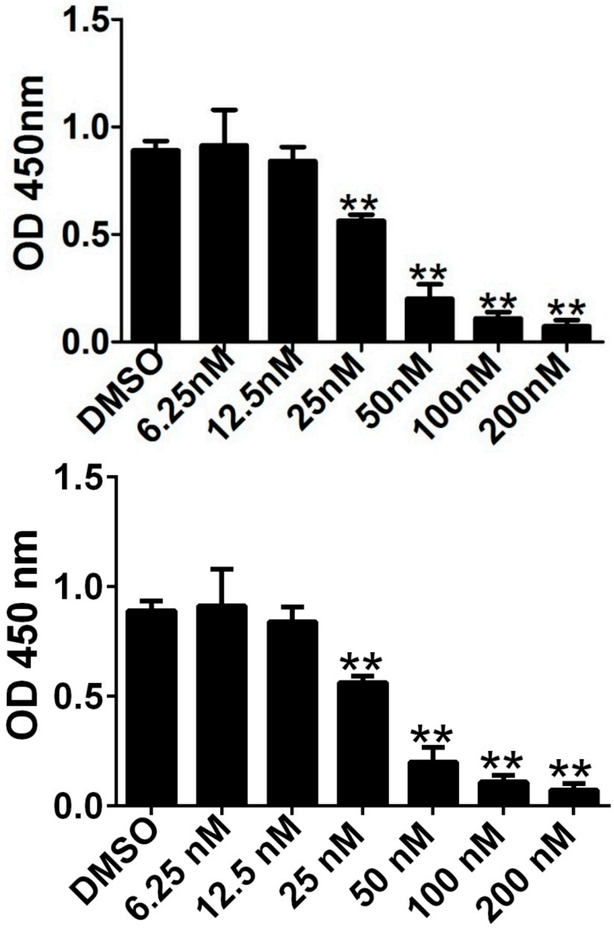

Figure S2. Cytotoxicity of TP on U937 cells. U937 cells were treated with different concentration of TP for 24 h, and their viability was determined using cell counting kit (CCK)-8 assay. ** $p < 0.01$ *versus* group without TP treatment. The data are presented as the mean ± SD from three independent experiments.

Table S1. The human target proteins of Triptolide (TP).

Name	GenInfo Identifier No.
nucleotide-binding oligomerization domain-containing protein 1	5174617
sphingosine 1-phosphate receptor 3	38788193
Chain A, Human Bcl2-A1 In Complex With Bim-Bh3 Peptide	167013344
Janus kinase 2	119579178
Tyrosyl-DNA phosphodiesterase1 protein	79154014
Sphingosine-1-phosphate receptor 4	15929025
serine/threonine kinase 33	12830367
Kruppel-like factor 5	124263658
